# The Malacosporean Myxozoan Parasite *Tetracapsuloides bryosalmonae*: A Threat to Wild Salmonids

**DOI:** 10.3390/pathogens9010016

**Published:** 2019-12-23

**Authors:** Arun Sudhagar, Gokhlesh Kumar, Mansour El-Matbouli

**Affiliations:** 1 Clinical Division of Fish Medicine, University of Veterinary Medicine, Vienna 1210, Austria; Arun.Sudhagar@vetmeduni.ac.at (A.S.); Mansour.El-Matbouli@vetmeduni.ac.at (M.E.-M.); 2 Central Institute of Fisheries Education, Rohtak Centre, Haryana 124411, India

**Keywords:** brown trout, bryozoan, climate change, myxozoan, proliferative kidney disease

## Abstract

*Tetracapsuloides bryosalmonae* is a myxozoan parasite responsible for proliferative kidney disease (PKD) in a wide range of salmonids. PKD, characterized by high mortality and morbidity, is well known for affecting aquaculture operations and wild salmonid populations across Europe and North America. The life cycle of *T. bryosalmonae* revolves around freshwater bryozoan and salmonid fish hosts. In recent years, *T. bryosalmonae* has been reported among wild salmonids from the European countries where it has not been reported previously. *T. bryosalmonae* is believed to be a possible reason for the diminishing wild salmonid populations in the natural water bodies of many European countries. Climate crisis driven rising water temperature can further accelerate the distribution of *T. bryosalmonae*. Expansion of the geographical distribution of *T. bryosalmonae* may further advocate the decline of wild salmonid populations, especially brown trout (*Salmo trutta*) in their habitats. Mathematical models are used to understand the pattern and distribution of *T. bryosalmonae* among the host in the natural water bodies. The present manuscript not only summarizes the incidences of *T. bryosalmonae* among the wild salmonid populations, but also discusses the contemporary understanding about the development of *T. bryosalmonae* in its hosts and the influences of various factors in the spread of the disease in the wild.

## 1. Introduction

**Figure 1:**
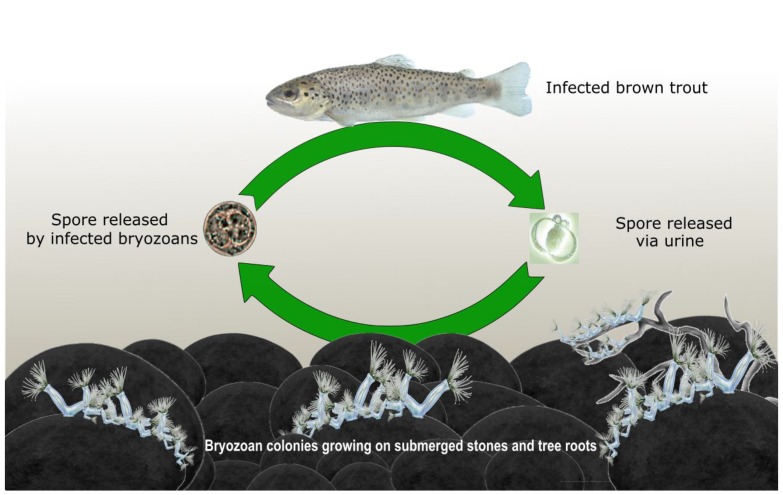
*Tetracapsuloides bryosalmonae* life cycle in salmonid fish host and invertebrate bryozoan host in wild environment. Climate change associated rising water temperature can enhance the proliferation of the parasite in both hosts. Waterfowls are suspected to be the long-distance vector of the parasite.

*Tetracapsuloides bryosalmonae* is a myxozoan parasite belonging to class Malacosporea and is well known for causing proliferative kidney disease (PKD) in salmonids [1]. The parasite life cycle exploits two different host species, an invertebrate bryozoan host and a vertebrate salmonid fish host (Figure 1) [2,3,4,5]. PKD in fish results in the enlargement of kidney and spleen, glomerulonephritis, anemia, ascites, exophthalmia, pale gills and darkened skin in the infected fish depending upon the severity. PKD alone can cause high mortality while secondary infections can speed up the death process and increase mortality even further [6]. *T. bryosalmonae* poses a serious threat to farmed and wild salmonids across Europe and North America. *T. bryosalmonae* is one of the major factors associated with the decline of endemic brown trout (*Salmo trutta*) population in the streams of Alps [7,8,9,10].

In recent years, *T. bryosalmonae* has been reported from new geographical locations from wild salmonids. This can be due to the spread of the parasite to new locations or due to the emergence of PKD outbreaks by the already existing parasite in that aquatic ecosystem unnoticed for a long period. However, *T. bryosalmonae* incidences are threatening as it can lead to massive fish kill events in natural water bodies affecting endemic fish population. *T. bryosalmonae* was responsible for the mass mortality of salmonids, particularly wild mountain whitefish (*Prosopium williamsoni*) from the Yellowstone river, Montana, USA [11]. This incidence led to the shutdown of 300 km river segment to public access for a certain period of time, which subsequently affected local tourism and the associated economy [12]. It is important to highlight that high-water temperature and low water flow cognate with this fish kill incidence.

The impact of *T. bryosalmonae* parasite on the protracted decline of wild fish populations is often more challenging to study as the infection dynamics is influenced by rising water temperature related to emerging climate change. Laboratory experiments depict that rising water temperature can enhance parasite production in both bryozoan and fish host [13,14,15,16]. Moreover, salmonids are cold water fish that are sensitive to higher temperatures, which may further aggravate the disease condition in the wild. Juvenile and adult salmonids prefer to occupy streams with temperature ranging between 13–18 °C and the water temperature approximately above 23–25 °C is fatal for salmonids [17]. Researchers also suspect waterfowl and common carp for the spread of *T. bryosalmonae* to naive locations. This review discusses about the *T. bryosalmonae* development in its host, emerging incidences of the parasite, role of climate change and the biological vectors that enhances the spread of the parasite among wild salmonids.

## 2. *Tetracapsuloides bryosalmonae*

Despite the fact that PKD like condition was first described in early 1920’s from Germany in rainbow trout (*Oncorhynchus mykiss*) [18], the disease condition was named as proliferative kidney disease in early 1970’s defining the main clinical features [19]. Transmission electron microscopic studies in 1985 revealed that a myxozoan parasite is the causative agent of PKD [20]. In the late 1990s, the involvement of an invertebrate bryozoan host was discovered in the life cycle of this myxozoan parasite [21]. The parasite was taxonomically distinguished from *Buddenbrockia* sp. and eventually named as *Tetracapsuloides bryosalmonae* under a new myxozoan class malacosporea [1,22,23]. Molecular phylogenetic studies suggest that myxozoans are evolutionarily primitive, morphologically degenerated cnidarians and their polar capsules are comparable with cnidarians nematocyst [24]. Two clades of *T. bryosalmonae* stemming from North America and Europe were identified. Interestingly, the North American clade of parasites was isolated from southern Europe. Scientific evidence suggests aquatic birds to be the possible vector for transporting the parasite between the continents [25].

**Figure 2:**
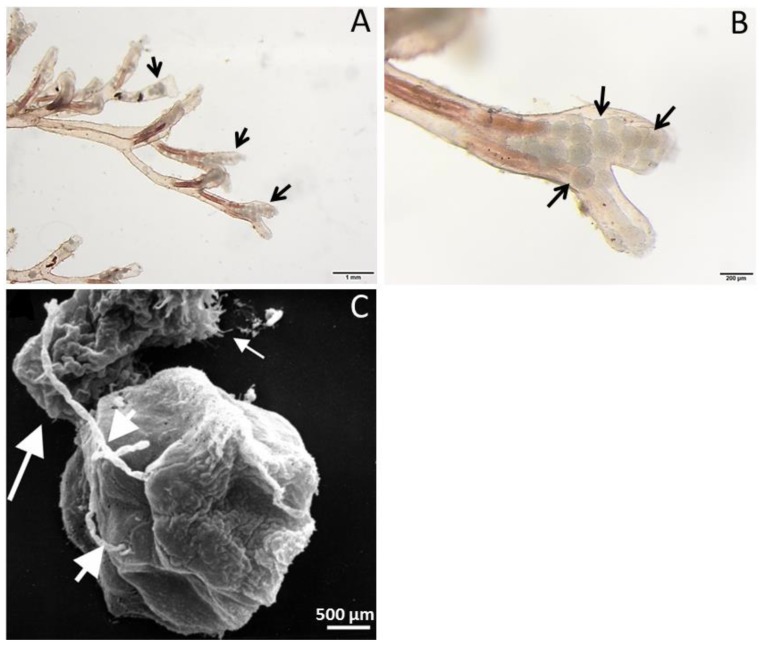
*Tetracapsuloides bryosalmonae* infection in bryozoan colonies and its scanning electron microscopy image. (**A**)—Laboratory-reared bryozoan *Fredericella sultana* colony infected with *T. bryosalmonae* and its body cavity filled with parasite sacs (arrows). (**B**)—Numerous mature parasite sacs (arrows) inside a bryozoan zooid that could readily release into the water and infect salmonids. (**C**)—Top view of a spore with its four polar capsules covered by valve cells. Short arrows: two polar filaments being fired. Large arrow: sporoplasm leaving the spore at the bottom side. Small arrow: formation of pseudopodia. The image was obtained from Grabner and El-Matbouli [5].

The freshwater bryozoan acts as a definitive host and the fish acts as an intermediate host for *T. bryosalmonae* [26]. Freshwater bryozoans commonly occur in rivers, ponds and lakes. They live in colonies on the surfaces of submerged rocks, plants, roots and branches of trees. Bryozoans are suspension feeders and their colonies propagate either by budding new zooids or by means of asexual reproductive seed-like structures called statoblasts. Statoblasts remain dormant and germinate to form a new colony under favorable conditions [27]. Bryozoan species of *Cristatella mucedo*, *Pectinatella magnifica*, *Plumatella rugosa*, *Plumatella* sp., and *Fredericella sultana* serve as hosts for *T. bryosalmonae* [28]. However, *F*. *sultana* is the most preferable host for *T. bryosalmonae* [29]. The route of parasite entry in the bryozoan host is not known but it is believed that the parasite might enter the host while the zooids feed on suspended materials in water [25]. After entry, the parasite goes into a cryptic stage developing a covert infection in the bryozoa. Following this, the parasite develops an overt infection by entering into a presaccualar stage (8–13 µm) which is observed swirling within the body cavity of the infected bryozoa [2,30]. Finally, the parasite develops to an amoeboid stage forming a prominent mature parasite spore sac (350 µm) having about 2800 to 4000 spores depending on size and packing density [22,31]. The mature spores having four polar capsules are then released by the infected bryozoan colonies, which infect salmonids (Figure 2).

**Figure 3:**
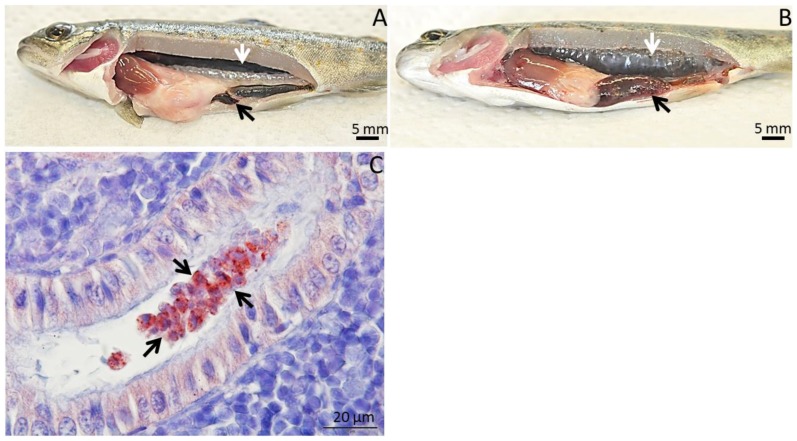
Clinical signs of proliferative kidney disease in brown trout. (**A**) Uninfected control brown trout shows normal kidney (white arrow) and spleen (black arrow). (**B**) *Tetracapsuloides bryosalmonae* infected brown trout shows clinical signs such as renal hypertrophy (white arrow) and splenomegaly (black arrow). (**C**) Intraluminal sporogonic parasite stages of *T. bryosalmonae* (arrow) inside the renal tubule of infected brown trout. Parasites were visualized by immunohistochemistry using anti-*T. bryosalmonae* monoclonal antibody and counterstained with hematoxylin (C image was obtained from Kumar et al. [35]).

Salmonids are the prime fish host for *T. bryosalmonae* infection. The parasite penetrates via gill epithelium of the fish and enters the circulatory system which disperses the parasite to various internal organs, principally to kidney [5]. Moreover, multiplication of extrasprogonic stages of parasite happens between the interstitium of kidney tissue and the extrasprogonic stages move to the lumen of kidney tubules where they differentiate into pseudoplasmodia. Single cellular amoeboid spore having two polar capsules develops within the pseudoplasmodia [20,32,33]. The developed parasites are then released via urine of infected fish that further infect the bryozoan host. The infected fish exhibits severe inflammatory responses leading to anemia, enlargement of kidney and spleen, consecutively leading to 100% mortality (Figure 3). Furthermore, granulomatous responses are often observed in chronic infection. Rainbow trout and brown trout are the most studied salmonid species to *T. bryosalmonae* infection [34]. Rainbow trout have not co-evolved with the European strain of *T. bryosalmonae* as infected rainbow trout cannot release mature parasite spores that can infect the bryozoan host. However, fish-to-bryozoan transmission is possible in brown trout and brook trout (*Salvelinus fontinalis*) [2,35,36]. In a recent study, brown trout recovered from PKD infection was able to release viable spores of *T. bryosalmonae* even after five years of infection [37]. This clearly demonstrates that brown trout recovered from PKD could act as a reservoir for *T. bryosalmonae* dispersal in the wild.

## 3. Geographic Distribution of *T. bryosalmonae* and Impact of PKD among Wild Salmonids

Apart from their endemic habitats, salmonids are distributed all across the globe by human intervention in the wild and captive environments. *T. bryosalmonae* has been reported from both farmed as well as wild salmonid populations in many countries of Europe and North America [6,38]. Even though pike (*Esox lucius*) has been reported for PKD by various investigators, the results are still questionable and have not been confirmed by molecular or ultrastructural evidence. With the development of better diagnostic techniques, the rapid expansion of the salmonid aquaculture industry, global warming and other anthropogenic activities, the geographical distribution of *T. bryosalmonae* is likely to expand [39,40,41]. Recent reports suggest that *T. bryosalmonae* may be associated with the decline of wild brown trout population in the European rivers, which has created more interest among researchers in the exploration of this parasite in wild salmonid populations [9,10,40,41,42,43,44,45,46,47]. Brown trout is endemic to Europe [48] and it is the widely reported fish species for *T. bryosalmonae* infection in the wild. Apart from brown trout, *T. bryosalmonae* has been reported from other salmonids from the wild (Table 1).

Early investigators speculated for the presence of infectious agent causing PKD in the rivers, which supply water to commercial aquaculture facilities with PKD outbreaks. Interestingly, PKD positive wild fish were found in those rivers [49,50,51,52,53]. Probably, the parasite might have spilled over from the wild to cultured salmonids held in aquaculture facilities. *T. bryosalmonae*-associated wild salmonid decline have been observed in many countries across northern hemisphere. However, *T. bryosalmonae* is one among many factors that are responsible for the wild salmonid decline. In most case, *bryosalmonae*-associated wild salmonid decline are protracted and mortalities are unnoticeable in the natural habitat. Early investigators used microscopical observations for the detection of the parasite and with the improvement of molecular diagnostic techniques, screening of *T. bryosalmonae* was possible even in cryptic infections. A wide survey was done in the early 2000s by sampling wild salmonids for *T. bryosalmonae* infection in the alpine rivers of Switzerland and the results of the study revealed that the geographical distribution of *T. bryosalmonae* could be much wider than anticipated [45]. From there on, more and more researchers explored the impact of *T. bryosalmonae* in wild salmonid populations and their association with salmonid stock depletion in European riverine systems [40,41,42,43,44,45,47,54,55]. *T. bryosalmonae* associated wild brown trout decline is well documented in Switzerland where *T. bryosalmonae*-positive sites were found all across the country [46,47]. Interestingly, *T. bryosalmonae* infections were widely observed in brown trout sampled below 800 m above the mean sea level. Furthermore, above this altitude, only a small amount of *T. bryosalmonae* positive brown trout were observed in the rivers drained by shallow lakes. This relationship between altitude and *T. bryosalmonae* incidence in wild brown trout could be associated with water temperature [45]. Evidence suggests that water temperature plays a pivotal role in affecting host–pathogen interaction during *T. bryosalmonae* infection in wild brown trout, thereby promoting the decline of their stocks in their habitat [56]. In addition, widespread distribution of bryozoan colonies covertly infected with *T. bryosalmonae* was observed from the rivers of England [57]. Comparison between wild brown trout and disease-free brown trout reared in the water enzootic for *T. bryosalmonae* showed that there were no prominent differences in disease prevalence between wild and captive trout [58]. Prevalence studies of renal myxosporidiosis in wild brown trout sampled from English rivers provided prevalence estimates for *T. bryosalmonae* and *Chloromyxum schurovi* [59]. Likewise in Austria, *T. bryosalmonae* is widely recorded among the wild brown trout population and could be a possible reason for their decline in the Austrian rivers [10].

A considerable reduction in wild Atlantic salmon (*Salmo salar*) population was observed in a Norwegian river due to *T. bryosalmonae* infection [55]. In a recent study, a very high prevalence of *T. bryosalmonae* was observed in Atlantic salmon, Arctic charr (*Salvelinus alpinus*) and brown trout from the rivers along the Norwegian coast [60]. Similarly, *T. bryosalmonae* incidences were associated with rising temperatures along with the decline of wild fish populations such as Arctic charr in Iceland [43]; brown trout and Atlantic salmon in Denmark [61]; pink salmon in Canada [62]; brown trout in Estonia [42,63]; brown and rainbow trout from Slovenia [64]; brown trout and grayling in Finland [65]. In a recent study, European whitefish (*Coregonus lavaretus*) infected with *T. bryosalmonae* were isolated from the lakes of Finland [66]. A detailed information of *T. bryosalmonae* reports from wild salmonids is presented in Table 1.

In the summer of 2016, a fish kill incident associated with *T. bryosalmonae* at Yellowstone river, Montana, USA affected local tourism, livelihood and recreational activity [11]. The initial estimates suggest a total economic output of the county reduced by about $359,750 to $523,815 [12]. The endemic salmonid, mountain whitefish were the most affected species in this incident. Local officials and anglers have reported a decline in the catch of mountain whitefish in the past decade and the reason for the catch reduction is unknown [67]. Detailed studies are needed to evaluate the catch decline of mountain whitefish keeping *T. bryosalmonae* as a prime suspect.

Apart from this, PKD has been reported in countries such as France [25,68], Italy [25,69,70], Spain [71], Ireland [72] and Czech Republic [73] from salmonid aquaculture facilities. Recently, a novel culture medium, Bryozoan Medium C was developed for the cultivation and maintenance of bryozoan host under laboratory conditions [74]. This may facilitate the maintenance of *T. bryosalmonae* parasite life cycle (Figure 1) collected from different geographical locations under laboratory condition for conducting further research. The resulting outcome will help in understanding the geographical origin, genetic differences, and infectivity of different strains of *T. bryosalmonae* parasite. Furthermore, environmental DNA (eDNA) monitoring [75,76] and lateral flow dipstick [77] based detection of *T. bryosalmonae* are valuable tools for pathogen surveillance in the wild.

## 4. Effect of Temperature on *T. bryosalmonae* Infected Bryozoan and Fish Host

In recent years, scientists have investigated the impact of climate change-associated temperature rise on infectious diseases that could significantly affect host–parasite relationship [56,82,83]. Scientific evidence collected from streams of Switzerland suggests that rising temperatures could expand the geographical distribution of *T. bryosalmonae* [45]. The rising water temperature can enhance the geographic distribution of *T. bryosalmonae* by enhancing the growth of bryozoan colonies thus more host biomass available for parasite life cycle [39], and similarly infected bryozoans and carrier fish could release more parasites at warmer temperature providing more infectious material to the environment for the spread [14,15,16].

Bryozoans tend to multiply and establish prominent colonies at higher water temperature (20 °C) [39]. In addition, higher water temperature (20 °C) tends to enhance overt infection and release of the parasite spores from the infected bryozoan colonies into the water bodies [16]. PKD outbreaks in fish are seasonal and are directly related to the water temperature. High temperature enhance *T. bryosalmonae* multiplication inside the fish host, and the clinical symptoms of PKD and associated mortalities are often observed when water temperature goes beyond 15 °C [6,13,14,15]. Brown trout reared at a water temperature of 15 °C showed a higher prevalence and intensity of parasites than trout maintained at 12 °C [84]. Rainbow trout exposed to water enzootic to *T. bryosalmonae* during winter months are found to be more resistant to PKD-associated mortalities in the following summer when the water temperature rises [85,86,87].

In recent years, *T. bryosalmonae* has been reported from northern latitudes, which may be affiliated with warmer waters [42,43,55,65]. In addition, it is possible that the parasite might have already been in those environments for a long time undiagnosed. For example, in a recent study archived samples of Sockeye salmon (*Oncorhynchus nerka*) reared in net-pens in a freshwater lake of Alaska collected in 1997 were found positive for *T. bryosalmonae* [80]. As the climatic patterns are expected to alter in the upcoming years, the geographical distribution of *T. bryosalmonae* might expand to newer regions. Apart from rising temperature, eutrophications of water bodies are often linked with *T. bryosalmonae* infection in the wild [38]. The availability of more food for bryozoans in eutrophic water bodies enhances their growth [31,88] which in turn facilitates higher host availability for parasite propagation.

Fish are cold-blooded (also referred to as ectothermic or poikilothermic) animals and their immune responses are temperature dependent [89,90,91,92]. Immune suppression associated with PKD due to the decrease of phagocytic activity by granulocytes is considered to cause a secondary infection in rainbow trout [93]. The swelling and proliferation of interstitial tissue of the kidney associated with PKD are due to immune responses by the fish against the pathogen. This immune reaction leads to the regression of kidney glomerulus and tubules, which may affect the osmoregulatory capacity and hematopoiesis of fish [14]. Infiltration of macrophages and necrotic cell debris are seen in the infected kidney [94,95]. Flow cytometry analysis showed the dominance of lymphocytes in the kidney of infected rainbow trout [93]. A significant B cell/antibody response and involvement of T helper cell activity are associated with the pathogenesis of PKD in rainbow trout [96,97]. Higher temperatures (18 °C) can influence the immune system of fish and can accelerate the clearance of *T. bryosalmonae* parasite in rainbow trout [94]. Laboratory experiments suggest that even a slight temperature difference may influence the immune strategy of the fish host against the parasite. At cooler temperatures (12 °C) the immune system of the rainbow trout host tends to tolerate the parasite, whereas a subtle increase of temperature (15 °C) redirects the host immune strategy towards resisting the parasite [96]. Studies on *T. bryosalmonae* infected brown trout from the wild showed that clinical symptoms of PKD are more strongly associated with kidney hyperplasia rather than with parasite load [98]. In addition to this, the defense strategy in different fish host varies, rainbow trout shows resistance whereas brown trout exhibits tolerance towards *T. bryosalmonae* [99]. Similarly, humoral immune response and endocytic pathway genes were downregulated profoundly in brown trout and, in contrast, they were upregulated in rainbow trout [100]. It is clear that temperature has a significant role in the selection of immune strategy of the fish hosts against *T. bryosalmonae*. In a recent experiment, protease genes involved in collagen catabolic process were downregulated during sporogenesis of *T. bryosalmonae* in brown trout [101]. Whereas, few collagen catabolic genes were upregulated in rainbow trout during recovery phase of PKD [102]. The collagen catabolic protease may play a crucial role in the persistence or clearance of *T. bryosalmonae* in brown trout and rainbow trout respectively. However, further studies on collagen catabolic protease are needed to gain insights on the difference in infection and co-evolution pattern of *T. bryosalmonae* observed between brown trout and rainbow trout.

## 5. Aquatic Birds and Common Carp as Vectors of *Tetracapsuloides bryosalmonae*

Waterfowls are well known to transport aquatic invertebrates and their eggs from one geographical location to another through both internal and external transportation [103]. Aquatic fowls could ingest *T. bryosalmonae*-infected statoblasts and introduce them into a naive habitat through their fecal matter or some statoblasts have hooks by which they attach themselves to the fowl feathers [2]. Phylogenetic studies hypothesize that the Northern American clade of *T. bryosalmonae* parasite might be transported to certain parts of Europe by waterfowls [25]. Besides, a recent laboratory experiment suggests that common carp (*Cyprinus carpio*) could also disperse infectious statoblasts by ingesting and subsequently excreting them [104]. Common carp are known to migrate up and down stream throughout the year up to 890 km [105]. However, they have different habitat preferences than brown trout. In fact, common carp typically dwell in lotic or slowly flowing waters, whereas brown trout prefer cold, lentic water bodies. Further studies are thereby needed to enhance our understanding of common carp and other potential fish species as vectors of statoblast carrying *T. bryosalmonae* in the wild. It is possible that aquatic fowl and some migratory fish species like common carp could be a potential vector, which aid in the geographical expansion of *T. bryosalmonae*. It is interesting to note that vertical transmission of *T. bryosalmonae* is possible through infected statoblasts of bryozoa and the parasite could remain in dormancy inside the statoblasts [106]. In addition, the changing climate pattern may possibly alter the movement of waterfowls which may further contribute to the spread of *T. bryosalmonae* [26].

## 6. Modelling Studies

Epidemiological models could be used effectively in predicting, preventing and controlling disease outbreaks [107]. Moreover, it may help government agencies to take policy decisions in cases of aquatic animal health management emergencies. The decline of native brown trout in the rivers of Switzerland was assessed using Bayesian probability network and this model aided in site-specific assessment of brown trout population [8]. This study used various variables such as gravel bed conditions, water quality, disease incidences including PKD, water temperature, habitat conditions, stocking practices, angler catch, and flood frequency. State–space models were developed for PKD aiming in understanding the spatio–temporal variation of *T. bryosalmonae* prevalences and fish abundance. This model could predict disease severity based on temperature regimes and could help with further understanding of the disease transmission [108]. The model was further improved into a comprehensive spatially predictive framework which could identify the key factors in propelling disease patterns in the wild fish population [109]. Case-study application of the developed model serves as an effective tool to predict and control the spread of disease in the wild [110].

Furthermore, a statistical model was employed in the evaluation of the factors yielding differences in relative parasite load and health damage among different *T. bryosalmonae* infected fish populations [63]. In this study, the disease severity was found to be higher in the warmer river than the colder one. Interestingly, the genetic variance for disease traits was higher in the brown trout dwelling in warmer river (maximum observed temperature 23.3 °C), whereas the genetic variance for parasite load was observed to be higher among the brown trout of cold river (maximum observed temperature 20.3 °C). Moreover, it is predicted that the variation in environmental factors may severely affect the evolution of the fish host towards the resistance and tolerance against the parasite.

## 7. Conclusions

In the past two decades, *T. bryosalmonae* has emerged as a threatening parasite that can decline wild salmonid populations. Routine population assessment of wild salmonids are necessary in the waters enzootic for *T. bryosalmonae* that may help to devise wild fish stock conservation strategies. PKD-recovered brown trout act as a carrier of *T. bryosalmonae* in the wild and shed the parasite for a very long period. This facilitates the *T. bryosalmonae* parasite to persist and establish itself in a wider geographical region. As aquatic fowl and common carp are hypothesized as vectors dispersing *T. bryosalmonae* parasite to different geographical locations, detailed interdisciplinary investigations are necessary to understand vector ecology, biology and impact of climate change on vector-borne transmission. *T. bryosalmonae*-infected trout are susceptible to secondary infection by opportunistic pathogens, which can even lead to mass mortality events in natural water bodies, particularly in the summer season. Temperature is known to influence the host–pathogen interaction of *T. bryosalmonae* parasite in its hosts. However, in natural ecosystems, climate change-driven stressors other than temperature as well as multiple-parasitism may also influence their interaction. Therefore, it is essential to conduct experiments to understand the influence of multiple factors and their interaction mimicking the natural environment on the host-parasite dynamics. Furthermore, next-generation sequencing based omics studies can help for the better understand of the host–parasite interaction and to explore the single nucleotide polymorphisms associated with the host resistance to the parasite. Likewise, it is necessary to characterize the strain variations of *T. bryosalmonae* parasite isolated from different geographical locations and study its interaction with different salmonid hosts. The screening of *T. bryosalmonae* using eDNA can serve as a promising tool to understand the abundance and distribution of the parasite in the riverine ecosystem. The host–parasite relationship of *T. bryosalmonae* is dynamic and influenced by various factors and it is essential to further explore the scientific possibilities to refrain the spread of the parasite among the wild fish population.

## Figures and Tables

**Table 1:** Report of *Tetracapsuloides bryosalmonae* infection from wild salmonids.

Reported Year	Fish Species	Country	Reference
1981	Grayling	United Kingdom	[49]
1982	Brown trout	United Kingdom	[50]
1984	Rainbow trout	USA	[52]
1991	Brown trout and grayling	United Kingdom	[51]
1992	Cutthroat trout	USA	[78]
1995	Kokanee salmon and chinook salmon	Canada	[53]
2002	Brown trout	United Kingdom	[79]
2002	Rainbow trout, brown trout and grayling	Switzerland	[54]
2004	Brown trout^+^ and cutthroat trout^++^	United Kingdom^+^, Switzerland^+^ and USA^++^	[25]
2007	Atlantic salmon	Norway	[55]
2007	Brown trout	Switzerland	[46]
2007	Brown trout	Switzerland	[47]
2008	Brown trout, rainbow trout and brook trout	Switzerland	[45]
2008	Brown trout	United Kingdom	[59]
2010	Arctic charr and brown trout	Iceland	[43]
2010	Brown trout and Atlantic salmon	Denmark	[61]
2010	Pink salmon	Canada	[62]
2013	Brown trout	Switzerland	[56]
2014	Brown trout	Estonia	[42]
2014	Brown trout and rainbow trout	Slovenia	[64]
2015	Brown trout	Switzerland	[58]
2016	Mountain white fish, rainbow trout, brown trout and cutthroat trout	USA	[11,12]
2016	Brown trout	Austria	[9]
2017	Brown trout and grayling	Finland	[65]
2017	Atlantic salmon, Arctic charr and brown trout	Norway	[60]
2017	Brown trout	Estonia	[63]
2018	Brown trout and rainbow trout	Austria	[10]
2018	European whitefish	Finland	[66]
2019	Chum salmon	USA (Alaska)	[80]
2019	Brown trout	Germany	[81]

^+^ and ^++^ corresponds to the fish group and the country on the same row.
